# Longitudinal changes in screen time, sleep, and sports/exercise activity in early adolescence

**DOI:** 10.1186/s12887-025-06368-z

**Published:** 2025-11-24

**Authors:** Jason M. Nagata, Christiane K. Helmer, Zain Memon, Sapna Ramappa, Jennifer H. Wong, Thang Diep, Abubakr A. Al-Shoaibi, Kyle T. Ganson, Alexander Testa, Fiona C. Baker, Kelley Pettee Gabriel, Erin E. Dooley

**Affiliations:** 1 Department of Pediatrics, University of California, Box 0503, 550 16th Street, 4th Floor, San Francisco, CA 94143 USA; 2 Factor-Inwentash Faculty of Social Work, University of Toronto, Toronto, ON Canada; 3 Department of Management, Policy and Community Health, University of Texas Health Science Center at Houston, Houston, TX USA; 4 Center for Health Sciences, SRI International, Menlo Park, CA USA; 5 Department of Epidemiology, University of Alabama at Birmingham, Birmingham, AL USA

**Keywords:** Screen time, Digital media, Sleep, Physical activity, Exercise, Adolescent, Longitudinal

## Abstract

**Background:**

This study aimed to examine longitudinal changes in screen time, sleep, and sports/exercise activity in early adolescents in the United States.

**Methods:**

We examined data over four annual assessments collected from the Adolescent Brain Cognitive Development (ABCD) Study (*N* = 9,519) from baseline (2016–2018, ages 9–10) to Year 3 (2019–2021, ages 12–13). Trends in screen time, sleep, sports/exercise activity, and other activities in a 24-hour time-use paradigm were derived from compositional data analysis, incorporating isometric log-ratio transformations and mixed-effects modeling.

**Results:**

Screen time increased by 0.23 h/day from baseline to Year 1, 1.58 h/day from baseline to Year 2, and 3.28 h/day from baseline to Year 3. Video game playing increased more in boys, and digital socializing increased more in girls. Sleep duration slightly decreased from baseline to Year 3. Sports/exercise activity slightly decreased from baseline to Year 1, then increased until Year 3. Time spent on other activities decreased by 0.13 h/day from baseline to Year 1, 1.60 h/day from baseline to Year 2, and 3.38 h/day from baseline to Year 3.

**Conclusions:**

Our findings indicate screen time may have increasingly displaced other activities as early adolescents age.

**Supplementary Information:**

The online version contains supplementary material available at 10.1186/s12887-025-06368-z.

## Introduction

Children’s and adolescents’ behaviors related to 24-hour movement cycles (e.g., physical activity, sedentary behaviors, and sleep duration) have been linked to physical, psychological, and educational outcomes [1]. Public health guidelines suggest at least an average of 60 min/day of physical activity across the week, no more than 2 h/day of screen time, and 9–12 h of sleep/night for 6–12-year-olds or 8–10 h of sleep/night for 13–17-year-olds [2, 3]. In 2019, however, fewer than 10% of U.S. children met these guidelines, with adherence decreasing with age [4].

Early adolescence is a critical point in development [5], during which poor sleep can impair overall well-being, academic performance, and mental and physical health [6]. Physical inactivity in adolescence is a risk factor for developing chronic diseases with increased morbidity and mortality in adulthood [7]. Elevated screen time in early adolescence is associated with negative mental health, greater behavioral problems, worse academic performance, and poorer sleep [8]. Our study is the first to examine longitudinal changes in screen time, sleep, and sports/exercise activity over four annual assessments in early adolescence. Notably, the period of observation in this study includes the COVID-19 pandemic [9]. A study using National Survey of Children’s Health (NSCH) data found that the prevalence of meeting youth guidelines for sleep increased in 2020 but decreased for physical activity and screen time—changes that may have been attributed to school closures and associated disruptions [10]. Using data from the Adolescent Brain Cognitive Development (ABCD) Study, we found that during the pandemic, adolescents’ daily step count decreased by 20.8% when compared to before the pandemic [11]. Similarly, we examined self-reported physical activity with 8.9% of adolescents meeting guidelines during the pandemic compared with 16.1% before the pandemic [12]. This decline may be explained by school closures, remote learning, and the cancellation of summer extracurricular activities [12]. However, it is unknown whether this decline in physical activity has persisted. Additionally, our previous work showed a drastic increase in screen time across a similar period from late childhood to early adolescence [13]. During the pandemic, average daily screen use was 7.7 h per day—nearly double the pre-pandemic average of 3.8 h per day [14]. This increase may be attributed to factors such as school closures and the use of screens as a coping mechanism for stress [14]. Previous research has also shown that adolescents experienced longer time in bed and later bedtimes during the COVID-19 pandemic, likely due to school closures and the resulting disruptions to daily routines [15]. Additionally, increased screen time was associated with later bedtimes and poorer sleep quality, including longer sleep onset latency and more frequent awakenings [15].

Given that time in one behavior is likely to offset time in another [16] and considering the shift by the World Health Organization (WHO) and other countries toward the adoption of 24-hour movement guidelines [17–21], it is important to longitudinally examine these behaviors in a 24-hour movement cycle approach. This approach is a relatively new paradigm that uses compositional data analysis (CoDA) techniques to examine the interdependent relationships between activity behaviors in a 24-hour day [22]. The current study uses CoDA to examine longitudinal changes that can reflect not only aging but also the effects of the pandemic on screen time, sleep, and sports/exercise activity. We hypothesized that screen time would increase across four years, whereas sleep and sports/exercise activity would decrease across that same period.

## Methods

This study examined four annual assessments (baseline: 2016–2018, ages 9–10 years; Year 1: 2017–2019; Year 2: 2018–2020; Year 3: 2019–2021) collected from the ABCD Study, a longitudinal cohort study focused on the health and cognitive development of a U.S. sample of 11,875 adolescents recruited from 21 study sites at baseline [23]. Participants were primarily recruited through schools, with efforts made to ensure diversity in gender, race and ethnicity, and socioeconomic status to reduce potential sampling bias [23]. More details about the ABCD Study can be found elsewhere [24]. Participants with missing screen time, sleep duration, sports/exercise activity, and sociodemographic data were excluded (*n* = 2356), resulting in a final sample of 9519 adolescents. Compared to included participants, excluded participants were more likely to be from racial or ethnic minorities, and to have parents with lower annual incomes and education (Table S1).

Parents/caregivers and adolescents provided informed consent and assent, respectively. The study was approved by the University of California, San Diego institutional review board (IRB) and local IRBs at each participating site.

Screen time was assessed using the ABCD Youth Screen Time Survey [25]. Adolescents answered 14 questions regarding the number of hours per weekday and weekend day spent engaging with various screen modalities (non-school related): viewing television (TV) shows or movies, watching videos (e.g., YouTube), playing video games, texting, video chatting (e.g., Skype, FaceTime), browsing the Internet, and using social media (e.g., Facebook, Instagram, Twitter). We divided screen time into three categories: TV shows or movies, video games (single and multi-player), and socializing (texting, video chatting, and social networking). To ensure that changes in screen time were not attributable to data availability only at certain years, we excluded Internet browsing (not recorded at baseline and Year 1) and video watching (not recorded at Year 3) from our screen time analysis for all four assessments. We calculated screen time across these subtypes for weekdays and weekends separately, then computed a weighted average to obtain participants’ average daily time in each subtype using the following formula: [(weekday average x 5) + (weekend average x 2)]/7 [26]. Screen time data were winsorized at 24 h. Self-reported screen time has been found to be significantly positively correlated (*r* = 0.49; *p* < 0.001) with an objective passively sensed screen use smartphone app [27]. A study involving adults reported a significant correlation between self-reported television viewing time and objectively measured viewing using an electronic monitor (Spearman ρ = 0.54, *p* < 0.001), with strong agreement—95% of reported values fell within 4 h of the average [28]. Comparable self-reported measures of television viewing have demonstrated acceptable test-retest reliability, with 7-day intraclass correlation coefficients ranging from 0.76 to 0.81 [29, 30].

Sleep duration was estimated using the parent-reported Sleep Disturbance Scale for Children (SDSC). Parents/caregivers were asked, “How many hours of sleep does your child get on most nights in the past six months?” with five options: 9–11 h, 8–9 h, 7–8 h, 5–7 h, and < 5 h per day. To facilitate analysis, we converted these categorical ranges into continuous estimates by assigning midpoint or estimated values of 10 h, 8.5 h, 7.5 h, 6 h, and 4 h, respectively. In the original sample used to develop SDSC, internal consistency was strong among control participants (α = 0.79) and remained acceptable in individuals with sleep disorders (α = 0.71). Test-retest reliability was also adequate for both total scores and individual items (*r* = 0.71) [31].

Sports/exercise activity duration was measured by the Sports Activity Involvement Questionnaire (SAI-Q) [23, 32]. The SAI-Q collected information on children’s participation in 23 different activities, including sports, music, and hobbies such as drawing. The SAI-Q includes items assessing whether a child’s participation in an activity occurred as part of a school-based or outside-of-school organized program, whether the child received private instruction, or whether the activity was self-taught. However, the questionnaire does not clearly distinguish between indoor vs. outdoor access for each activity. It was completed by parents or caregivers at baseline and during each follow-up year. At baseline, they reported whether their child had ever participated in each activity, and at follow-ups, they indicated whether the child had engaged in each activity since the previous visit (yes/no). Example questions include, “Has your child participated in surfing?” and for each activity, “Since we last saw you, about how many months did your child participate in this activity?” For each activity, parents/caregivers were asked to report (1) time spent (mins) per session (tspent), (2) number of days per week participating (perwk), (3) number of months per year participating (nmonth), and (4) whether the child has participated in their lifetime at baseline or since last visit for follow-up years (yes/no). For each sport, the mean hours per day of participation was calculated using the following formula: (tspent*perwk*nmonth*4.33)/(52*60*7), where 4.33 represents the number of weeks per month. This value was then totaled across sports to compute the total mean hours per day of sports/exercise activity duration.

The three categories (screen time, sleep duration, and sports/exercise activity) examined did not necessarily capture all activities in a full 24-hour day. Thus, an “other activities” category was calculated by subtracting total time spent on screen time, physical activity, and sleep from 24 h. The data in this category were winsorized at 0 h. This category may capture less salient light-intensity activities such as family interactions, in-person social activities, self-care, chores, and schoolwork (including using a device) that are challenging to recall and report accurately via questionnaire, as well as modalities of screen time not captured by the ABCD Youth Screen Time Survey.

We analyzed the change in daily movement behaviors (screen time, sleep duration, sports/exercise activity, and other activities) over four years. We used CoDA, which used geometric means, to account for the nature of our time-varying data. This method ensures valid inferences by respecting the constant-sum constraint of 24-hour time-use compositions. We applied isometric log-ratio (ILR) transformations to express the behaviors in a format suitable for regression. Each ILR coordinate compares one behavior (e.g., sleep) to the rest of the composition using a method called pivot coordinates.

We then fit mixed-effects models for each ILR coordinate, with time as the independent variable. The mixed-effects models included a random intercept for participant ID to account for individual-level variability. However, random slopes for time were not included. Models were adjusted for age and data collection period (e.g., before and during the COVID-19 pandemic) as time-varying covariates for all study years. In our analysis, each participant’s assessment was individually coded as pre-COVID-19 if it was before March 13th, 2020) or during COVID-19 if it was between March 13th, 2020, and December 31 st, 2021. Accordingly, some data from Years 2 and 3 may have been collected during the pandemic and potentially influenced by factors such as school closures and remote learning. Data collected in 2022 and beyond were not included in the analysis; thus, post-pandemic recovery periods, including widespread school reopening, were not accounted for. We also adjusted for baseline variables of sex, race and ethnicity, study site, parental education, and household income. Model predictions were back-transformed into daily hours using inverse ILR transformation and normalized to sum to 24 h. Finally, we used bootstrapping with 1000 resamples to generate 95% confidence intervals for predicted mean behavior hours and for change-from-baseline estimates over time. Sex-stratified analyses were conducted for mean behavior hours and change-from-baseline estimates. All analyses were performed in R using the following packages: compositions, zCompositions, lme4, and ggtern.

## Results

Our analytical sample included 9,519 adolescents (mean [SD] age, 9.9 [0.6] years; 47.7% female) from baseline (2016–2018, ages 9–10) to Year 3 (2019–2021, ages 11–14) (Table 1). Participants in this study were racially diverse (Asian, 6.2%; Black, 17.4%; Latino or Hispanic, 16.2%; Native American, 3.5%; White, 55.5%; other, 0.9%). Regarding household income, 54.6% reported earning <$75,000, while 45.4% reported earning ≥ $75,000. Most parents had higher levels of education, with 85.8% completing a college education or more and 14.2% completing high school education or less.

**Table 1:** Sociodemographic characteristics of Adolescent Brain Cognitive Development (ABCD) Study participants at baseline (2016–2018) (*N* = 9519)

Sociodemographic characteristics	Mean (SD)/*n* (%)
Age (years)	9.9 (0.6)
Sex, n (%)	
Female	4537 (47.7%)
Male	4981 (52.3%)
Race and ethnicity, n (%)	
Asian	586 (6.2%)
Black	1659 (17.4%)
Latino/Hispanic	1545 (16.2%)
Native American	335 (3.5%)
Other	85 (0.9%)
White	5282 (55.5%)
Household income, n (%)	
$24,999 or less	1069 (11.2%)
$25,000 to $49,999	1224 (12.9%)
$50,000 to $74,999	2899 (30.5%)
$75,000 to $99,999	1350 (14.2%)
$100,000 to $199,999	2899 (30.5%)
$200,000 and greater	1056 (11.1%)
Parent’s highest education, n (%)	
High school education or less	1346 (14.2%)
College education or more	8162 (85.8%)

**Fig. 1:**
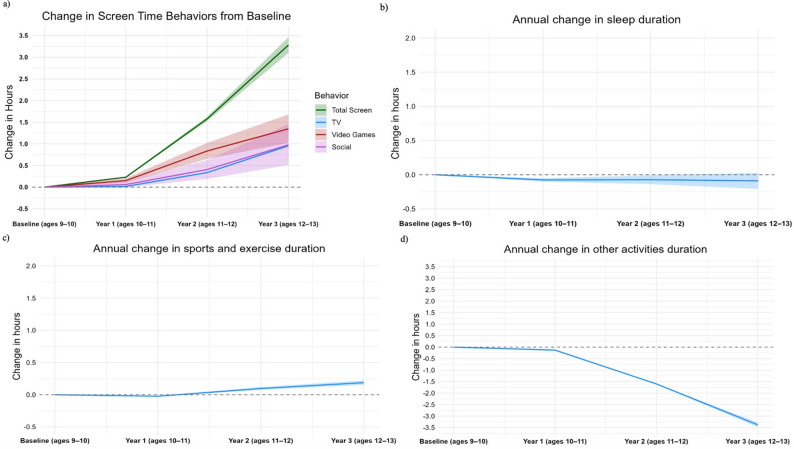
Bootstrapped annual mean change (hours/day) for (a) screen time, (b) sleep, (c) sports/exercise activity, and (d) other activity

Figure 1 shows bootstrapped trends in screen time, sleep, sports/exercise activity, and time spent on other activities in a 24-hour time-use paradigm in early adolescents from baseline (2016–2018) to Year 3 (2019–2021) assessments. Sleep duration slightly decreased from baseline to Year 3. Sports/exercise activity slightly decreased from baseline to Year 1, then increased until Year 3. Screen time and time spent on other activities substantially changed, with specific trends detailed subsequently.

**Fig. 2:**
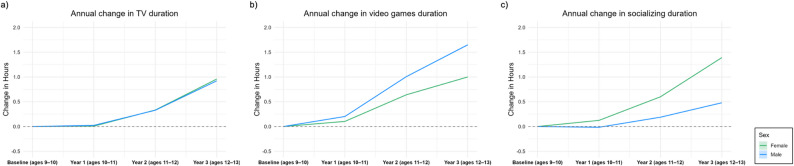
Boot-strapped annual mean change (hours/day) stratified by sex: (**a**) TV shows or movies, (**b**) video games, and (**c**) socializing

Table 2 shows the average time spent (hours/day) on various movement behaviors from baseline to Year 3 in the overall and sex-stratified samples. From the results of the mixed effects models, screen time increased by 0.23 h/day from baseline to Year 1 (95% CI: 0.22, 0.24, *p* < 0.001), 1.58 h/day from baseline to Year 2 (95% CI: 1.53, 1.63, *p* < 0.001), and 3.28 h/day from baseline to Year 3 (95% CI: 3.10, 3.46, *p* < 0.001) (Table S2). Each screen activity type also increased over the four assessments. Digital socializing was consistently one of the lowest out of the three screen activity modalities but increased by, on average, 0.98 h/day from baseline to Year 3 (95% CI: 0.51, 1.45, *p* < 0.001). Time spent on other activities decreased by 0.13 h/day from baseline to Year 1 (95% CI: −0.16, −0.10, *p* < 0.001), 1.60 h/day from baseline to Year 2 (95% CI: −1.61, −1.59, *p* < 0.001), and 3.38 h/day from baseline to Year 3 (95% CI: −3.48, −3.28, *p* < 0.001). Figure 2 shows boot-strapped sex-stratified trends in screen time, though the confidence intervals are very narrow. From baseline to Year 3, TV show or movie viewing increased by 0.96 h/day for females (95% CI: 0.95, 0.96, *p* < 0.001) and 0.92 h/day for males (95% CI: 0.92, 0.93, *p* < 0.001), video game playing increased by 1.00 h/day for females (95% CI: 1.00, 1.01, *p* < 0.001) and 1.65 h/day for males (95% CI: 1.64, 1.66, *p* < 0.001), and digital socializing increased by 1.39 h/day for females (95% CI: 1.38, 1.40, *p* < 0.001) and 0.480 h/day for males (95% CI: 0.478, 0.481, *p* < 0.001) (Table S2).

**Table 2:** Adjusted mean time spent on various movement behaviors in the Adolescent Brain Cognitive Development (ABCD) Study from baseline to year 3, overall and stratified by sex (*N* = 9,519)

Overall					Female				Male			
Activity (hours)	Baseline	Year 1	Year 2	Year 3	Baseline	Year 1	Year 2	Year 3	Baseline	Year 1	Year 2	Year 3
Total screen time	2.86	3.09	4.44	6.15	2.63	2.87	4.25	6.08	3.10	3.31	4.63	6.21
TV shows or movies	0.99	1.01	1.33	1.95	0.97	0.98	1.31	1.94	1.02	1.04	1.35	1.96
Video games	0.89	1.04	1.72	2.24	0.76	0.86	1.42	1.79	1.02	1.22	2.03	2.68
Digital socializing	0.98	1.04	1.39	1.96	0.90	1.03	1.52	2.35	1.06	1.05	1.25	1.57
Sleep	9.16	9.08	9.09	9.07	9.15	9.10	9.02	8.95	9.17	9.07	9.15	9.18
Sports/exercise activity	0.60	0.57	0.69	0.78	0.60	0.57	0.71	0.82	0.60	0.58	0.67	0.75
Other activity	11.38	11.25	9.78	8.00	11.62	11.46	10.02	8.15	11.14	11.04	9.54	7.85

Means were estimated from mixed-effects models using 24-hour movement behavior compositions

We tested the interaction of time and sex across each ILR-transformed model to identify sex-specific longitudinal patterns. There were significant interactions in [ilr1, ilr2, ilr3, and ilr5] suggesting differential shifts in behavioral balances between males and females, while non-significant effects in [ilr4] imply comparable trajectories. Because ILR coordinates are mathematical balances of multiple behaviors, these interaction tests indicate sex differences in the overall 24-h composition but do not correspond directly to any single behavior.

## Discussion

In this demographically diverse, national sample of U.S. adolescents aged 9–10 years old at baseline and 12–13 years old at Year 3, we found an increase in the viewing of TV shows and movies, playing video games, and digital socializing across the years. Further, sleep duration and time spent on other activities decreased whereas the duration of sports/exercise activity slightly decreased from baseline to Year 1, then increased until Year 3. Additionally, males had a higher increase in time spent playing video games, while females experienced a higher increase in time spent digitally socializing since baseline.

Screen time drastically increased over time with a more than a 3-hour/day increase across three years, consistent with the significant increase shown in our previous work [13]. This could reflect the effects of peer pressure, less parental supervision of adolescent screen time, and increased use of screens for daily activities during the COVID-19 pandemic [33]. Previous research from the ABCD Study has shown that each additional hour of screen time is associated with higher BMI, as well as higher odds of diabetes and elevated waist circumference [34]. Given that screen time increased by more than three hours per day across the years, including the COVID-19 pandemic, adolescents may be at heightened risk for cardiometabolic disease. Screen time increased, while sleep and time spent on other activities decreased across the years. Considering the timeframe includes the COVID-19 pandemic, the significant decrease in time spent on other activities, which may include in-person socialization and unstructured family or peer interactions, may explain the increase in screen time due to pandemic-related restrictions. Screen time spent on socializing was consistently one of the lowest among the screen time activity types but showed a 1-hour/day increase from baseline to Year 3. The increase in digital socializing may explain the decreasing levels of sleep, as social media use has been linked with lower odds of sleep [35, 36]. Additionally, we found that boys spent more time playing video games, while girls spent more time digitally socializing, consistent with our previous work [37]. Moreover, the increase in video game time was greater for boys, while the increase in digital socializing was greater for girls across the years.

Sleep duration decreased over the four annual assessments, which was similarly reflected in an NSCH study using 2016–2017 data, which reported that the prevalence of meeting sleep guidelines significantly declined from ages 6 to 17 years [4]. However, an ABCD study found that sleep duration fluctuated during the pandemic, with an increase during May–August 2020, partly due to summer break, but declined in October 2020 to levels lower than pre-pandemic [15]. One possible explanation for the relatively small decline in sleep duration observed in our study is that the COVID-19 pandemic may have disrupted school schedules and daily routines, leading to later sleep and wake times and greater opportunities for sleep [15].

We found sports/exercise activity to slightly decrease from baseline to Year 1, then increase until Year 3, yet sports/exercise activity remained below the age recommendations of 60 min of daily physical activity [38]. The slight increase in sports/exercise activity during the COVID-19 pandemic may be partially explained by increased indoor play, which may include playing cards, drawing/painting, and doing arts and crafts, due to the lockdown requirements [39].

Our findings have important clinical and policy implications. Parents should continue to vigilantly monitor adolescent screen time as adolescents age to prevent potential displacement of physical activity and sleep. Pediatricians should offer counseling that includes a more focused discussion on the use of video games for boys and digital socializing for girls. School administrators should also explore new ways to promote physical activity within schools, such as supporting active travel to school (e.g., Safe Routes to School programs) and integrating physical education into adolescent’s digital media interests such as education tools or digital health solutions, like apps that leverage screen time with physical activity content. Future studies can also extend the follow-up period to assess trends in screen time, sleep, and physical activity among older adolescents. Additionally, future research could leverage state-level variation in COVID-19 policies to examine how structural factors shaped children’s screen use, sleep, and physical activity. Such analyses may help disentangle the impact of policy environments from individual- or family-level influences during the pandemic.

This study has several limitations. The ABCD SAI-Q posed different questions at baseline compared to follow-up years, as the baseline questionnaire asked how many months the child participated in the activity during their most active period in their lifetime while the follow-up questionnaire asked how many months the child participated in the past year. To address this, we estimated average daily sports/exercise activity duration over each year. While this approach helped standardize the measures across time points, some residual bias is likely, and baseline sports/exercise activity may still be overestimated, potentially influencing the observed longitudinal trends. The SAI-Q has also not been formally validated; however, the items demonstrate face and content validity, as they align well with commonly recognized categories of youth activities. In addition, the SAI-Q does not delineate between indoor and outdoor activities, and our analyses did not differentiate among activity types due to the large breadth of activities included in the questionnaire. Another limitation was converting categorical sleep duration to continuous estimates in the SDSC, which may introduce measurement error. When interpreting longitudinal data, it is also a challenge to fully disentangle the effects of age, period of measurement, and cohort on outcomes. While CoDA appropriately models the relative composition of outcomes, it does not resolve the age-period-cohort (APC) problem or fully separate the effects of age, period, and cohort. Our analysis uses two methods to address these challenges. First, the purpose of CoDA is to correctly model our outcome variable, using the 24-hour time-use composition. This transformation is necessary to meet the assumptions of linear regression when dealing with constant-sum data. Separately, we addressed the APC issue through our mixed-effects model specification. The model simultaneously accounts for the age effect (with age as a time-varying covariate), a major period effect (the COVID-19 pandemic), and stable inter-individual differences, including cohort (with a random intercept for each subject). While these methods help address APC confounding, they do not fully resolve the APC problem. Another limitation was the use of parent-reported sports/exercise activities and sleep, which may be less reliable than child report. Adolescents were not asked about specific video game types, which could be an area of future research. Additionally, because Internet browsing and video watching were not recorded across all four years and were excluded from our analysis and because non-school related screen time was excluded from recreational screen time, any time spent on these activities would fall under “other activities.” As a result, this category is not well-defined, as it may include unmeasured screen use alongside non-screen activities, such as school and family commitments, preventing us from fully understanding how participants spent their time. The survey measures did not specify whether data assessments reflected term time or holidays, which may have introduced seasonal variation in reported behaviors [40]. Lastly, the analytic sample may not be fully representative of the U.S. population, as it includes a higher proportion of White participants and participants whose parents have a higher education and income, and a lower proportion of individuals from Latino, Native American, and lower-income backgrounds.

Despite these limitations, this study has several strengths. While previous studies have examined longitudinal changes in screen time, sleep, and physical activity in adolescence, ours is the first to detect changes in these activities using CoDA with four annual assessments, while also investigating different subtypes of screen time. Additionally, we used data from a large, national, and demographically diverse prospective cohort, increasing the generalizability of our findings.

## Conclusion

In conclusion, our longitudinal study detected changes in screen time, sleep, and sports/exercise activity over four annual assessments in early adolescence. Our findings indicate that screen time increases while time spent engaging in other activities decreases as early adolescents aged, offering insights for current guidance.

## Supplementary Information

Additional file 1. Table S1, Table S2.

## Data Availability

Data used in the preparation of this article were obtained from the ABCD Study ([https://abcdstudy.org](https:/abcdstudy.org)), held in the NIH Brain Development Cohorts (NBDC) Portal.

## Abbreviations

ABCD: Adolescent Brain Cognitive Development Study
APC: Age-period-cohort
CoDA: Compositional data analysis
ILR: Isometric log-ratio transformations
SDSC: Sleep Disturbance Scale for Children
SAI-Q: Sports Activity Involvement Questionnaire
TV: Television
