# Supplementary material for: Longitudinal changes in screen time, sleep, and sports/exercise activity in early adolescence
Source: BMC Pediatr. 2025 Nov 24;26:109. doi: 10.1186/s12887-025-06368-z (PMC12903580; doi:10.1186/s12887-025-06368-z)
Supplement: Supplementary file 1 — Additional file 1. Table S1, Table S2. [file 12887_2025_6368_MOESM1_ESM.docx]

| Table S1. Comparison of baseline characteristics between included and excluded participants. | | | |
| --- | --- | --- | --- |
|  | Included | Excluded |  |
|  | N=9,519 | N=2,356 |  |
| Sociodemographic characteristics | Mean (SD) / % | Mean (SD) / % | p-value |
| Age (years) | 9.9 (±0.6) | 9.9 (±0.6) | 0.245 |
| Sex (%) |  |  |  |
| Female | 47.7% | 47.7% | 0.845 |
| Male | 52.3% | 52.3% |  |
| Race and ethnicity (%) |  |  |  |
| Asian | 6.2% | 6.1% | <0.001 |
| Black | 11.4% | 30.0% |  |
| Latino/Hispanic | 16.2% | 21.1% |  |
| Native American | 3.5% | 3.4% |  |
| White | 55.5% | 1.2% |  |
| Other | 0.9% | 38.3% |  |
| Household income (%) |  |  |  |
| Less than $25,000 | 11.2% | 26.7% | <0.001 |
| $25,000 through $49,999 | 12.9% | 17.4% |  |
| $50,000 through $74,999 | 30.5% | 12.5% |  |
| $75,000 through $99,999 | 14.2% | 11.1% |  |
| $100,000 through $199,999 | 30.5% | 22.2% |  |
| $200,000 and greater | 11.1% | 10.1% |  |
| Parent's highest education (%) |  |  |  |
| High school education or less | 14.2% | 29.7% | <0.001 |
| College education or more | 85.8% | 70.3% | <0.001 |

| Table S2. Annual changes in screen time, sleep, sports/exercise activity, and other activities in the Adolescent Brain Cognitive Development (ABCD) Study, overall and stratified by sex. | | | | | | | | | |  |
| --- | --- | --- | --- | --- | --- | --- | --- | --- | --- | --- |
|  |  | Overall | | | Female | | | Male | | |
| Time | Behavior | Change in the mean (h/day) | 95% CI | p-value | Change in the mean (h/day) | 95% CI | p-value | Change in the mean (h/day) | 95% CI | p-value |
| Baseline to Year 1 | Total screen time | 0.23 | (0.22, 0.24) | **<0.001** | 0.24 | (0.23, 0.24) | **<0.001** | 0.210 | (0.208, 0.212) | **<0.001** |
|  | TV shows or movies | 0.02 | (0.01, 0.02) | **<0.001** | 0.008 | (0.0077, 0.0081) | **<0.001** | 0.023 | (0.023, 0.024) | **<0.001** |
|  | Video games | 0.15 | (0.10, 0.20) | **<0.001** | 0.103 | (0.102, 0.104) | **<0.001** | 0.202 | (0.200, 0.204) | **<0.001** |
|  | Digital socializing | 0.06 | (-0.01, 0.13) | 0.806 | 0.12 | (0.12, 0.13) | **<0.001** | -0.015 | (-0.016, -0.015) | **<0.001** |
|  | Sleep | -0.08 | (-0.10, -0.05) | **<0.001** | -0.064 | (-0.064, -0.063) | **<0.001** | -0.094 | (-0.095, -0.093) | **<0.001** |
|  | Sports/exercise activity | -0.02 | (-0.03, -0.02) | **<0.001** | -0.0240 | (-0.0243, -0.0237) | **<0.001** | -0.0231 | (-0.0234, -0.0228) | **<0.001** |
|  | Other activities | -0.13 | (-0.16, -0.10) | **<0.001** | -0.147 | (-0.149, -0.146) | **<0.001** | -0.093 | (-0.094, -0.092) | **<0.001** |
| Baseline to Year 2 | Total screen time | 1.58 | (1.53, 1.63) | **<0.001** | 1.57 | (1.56, 1.58) | **<0.001** | 1.52 | (1.51, 1.53) | **<0.001** |
|  | TV shows or movies | 0.34 | (0.33, 0.35) | **<0.001** | 0.33 | (0.33, 0.34) | **<0.001** | 0.329 | (0.327, 0.330) | **<0.001** |
|  | Video games | 0.83 | (0.65, 1.02) | **<0.001** | 0.640 | (0.637, 0.644) | **<0.001** | 1.01 | (1.00, 1.01) | **<0.001** |
|  | Digital socializing | 0.41 | (0.19, 0.62) | **<0.001** | 0.60 | (0.59, 0.61) | **<0.001** | 0.188 | (0.187, 0.189) | **<0.001** |
|  | Sleep | -0.07 | (-0.14, 0.01) | **<0.001** | -0.13 | (-0.14, -0.13) | **<0.001** | -0.004 | (-0.012, 0.003) | 0.194 |
|  | Sports/exercise activity | 0.10 | (0.07, 0.12) | **<0.001** | 0.107 | (0.106, 0.108) | **<0.001** | 0.072 | (0.071, 0.072) | **<0.001** |
|  | Other activities | -1.60 | (-1.61, -1.59) | **<0.001** | -1.55 | (-1.56, -1.54) | **<0.001** | -1.59 | (-1.60, -1.58) | **<0.001** |
| Baseline to Year 3 | Total screen time | 3.28 | (3.10, 3.46) | **<0.001** | 3.35 | (3.33, 3.37) | **<0.001** | 3.05 | (3.03, 3.07) | **<0.001** |
|  | TV shows or movies | 0.96 | (0.93, 0.98) | **<0.001** | 0.96 | (0.95, 0.96) | **<0.001** | 0.92 | (0.92, 0.93) | **<0.001** |
|  | Video games | 1.35 | (1.02, 1.68) | **<0.001** | 1.00 | (1.00, 1.01) | **<0.001** | 1.65 | (1.64, 1.66) | **<0.001** |
|  | Digital socializing | 0.98 | (0.51, 1.45) | **<0.001** | 1.39 | (1.38, 1.40) | **<0.001** | 0.480 | (0.478, 0.481) | **<0.001** |
|  | Sleep | -0.09 | (-0.21, 0.03) | 0.999 | -0.19 | (-0.20, -0.17) | **<0.001** | 0.03 | (0.02, 0.05) | **<0.001** |
|  | Sports/exercise activity | 0.19 | (0.15, 0.22) | **<0.001** | 0.20 | (0.20, 0.21) | **<0.001** | 0.134 | (0.133, 0.135) | **<0.001** |
|  | Other activities | -3.38 | (-3.48, -3.28) | **<0.001** | -3.37 | (-3.38, -3.36) | **<0.001** | -3.22 | (-3.23, -3.21) | **<0.001** |
